# Gut Microbial Adaptation to Varied Altitudes and Temperatures in Tibetan Plateau Yaks

**DOI:** 10.3390/microorganisms12071350

**Published:** 2024-07-01

**Authors:** Yanbin Zhu, Jia Wang, Yangji Cidan, Hongzhuang Wang, Kun Li, Wangdui Basang

**Affiliations:** 1 Institute of Animal Husbandry and Veterinary Medicine, Tibet Academy of Agriculture and Animal Husbandry Sciences, Lhasa 850009, China; zhuyanbin126@126.com (Y.Z.); 13889092363@163.com (Y.C.); wanghongzhuang66@163.com (H.W.); 2 Linzhou Animal Husbandry and Veterinary Station, Lhasa 850009, China; 3 College of Veterinary Medicine, Gansu Agricultural University, Lanzhou 730070, China; 4 College of Veterinary Medicine, Nanjing Agricultural University, Nanjing 210095, China; 17119103@njau.edu.cn

**Keywords:** Tibetan plateau yak, intestinal microbiome, ecological resilience, next-generation sequencing

## Abstract

The yak (*Bos grunniens*) exhibits exceptional regional adaptability, enabling it to thrive in the distinctive ecological niches of the Qinghai–Tibet Plateau. Its survival relies on the intricate balance of its intestinal microbiome, essential for adapting to harsh environmental conditions. Despite the documented significance of bacteria and fungi in maintaining intestinal homeostasis and supporting immune functions, there is still a substantial gap in understanding how the composition and functionality of yak gut microbiota vary along altitude–temperature gradients. This study aims to fill this gap by employing 16S rRNA and ITS amplicon sequencing techniques to analyze and compare the intestinal microbiome of yaks residing at different elevations and exposed to varying temperatures. The findings demonstrate subtle variations in the diversity of intestinal bacteria and fungi, accompanied by significant changes in taxonomic composition across various altitudes and temperature gradients. Notably, Firmicutes, Actinobacteriota, and Bacteroidota emerged as the dominant phyla across all groups, with Actinobacteriota exhibiting the highest proportion (35.77%) in the LZF group. Functional prediction analysis revealed significant associations between the LZF group and metabolic pathways related to amino acid metabolism and biosynthesis. This suggests a potential role for actinomycetes in enhancing nutrient absorption and metabolism in yaks. Furthermore, our findings suggest that the microbiota of yaks may enhance energy metabolism and catabolism by modulating the Firmicutes-to-Bacteroidota ratio, potentially mitigating the effects of temperature variations. Variations in gut bacterial and fungal communities among three distinct groups were analyzed using metagenomic techniques. Our findings indicate that microbial genera exhibiting significant increases in yaks at lower altitudes are largely beneficial. To sum up, our research investigated the changes in gut bacterial and fungal populations of yaks residing across diverse altitude and temperature ranges. Moreover, these results enhance comprehension of gut microbial makeup and variability, offering perspectives on the environmental resilience of dry lot feeding yaks from a microbial angle.

## 1. Introduction

The ruminant microbiota, comprising thousands of microorganisms, plays a crucial role in diverse physiological functions, including nutrient absorption, material metabolism, energy supply, body growth, and immune regulation [1,2,3]. Bacteria constitute up to 98% of the gut microbiome, while the remaining 2% comprises fungi, viruses, protozoa, and other microorganisms [4]. Although bacteria, which play a crucial role in regulating mucosal immunity and enhancing gastrointestinal health, dominate the gut microbiome, the importance of gut fungi is increasingly being recognized despite their lower abundance compared to bacteria [5,6]. These fungi significantly impact intestinal flora homeostasis, immune system development, and inflammatory reactions [7,8]. The git microbiome constitutes a dynamic and complex ecosystem [9]. Dysfunctions, such as those induced by environmental pollution, pathogen infection, or immune disorders, can lead to aberrant metabolism and interfere with host growth and development [10,11]. For instance, many investigations have pinpointed intestinal dysbiosis as a major factor contributing to enteric inflammation in conditions such as Crohn’s disease (CD), inflammatory bowel disease (IBD), and ulcerative colitis (UC), alongside its detrimental impacts on the digestive system [12]. Furthermore, gut microbial dysbiosis can impair the function of other organs, such as the liver [13,14]. Additionally, it is considered a potential aggravating or predisposing factor in various neurological autoimmune conditions and metabolic disorders [15,16].

The yak (*Bos grunniens*) prospers in the unique ecological niches of the Qinghai–Tibet Plateau due to its exceptional regional adaptability. This unique environment is characterized by harsh climates, including low temperatures, high altitudes (with an average above 4000 m), low oxygen levels (pO2), and intense ultraviolet (UV) radiation [17,18,19]. In China, there are around 16 million yaks, constituting more than 90% of the global yak population and roughly 20% of the entire cattle population in the country. Yaks hold an irreplaceable ecological, social, and economic status, providing essential resources for Tibetans and other nomadic pastoralists living in high-altitude environments [18]. Owing to distinctive geographical settings and cultural traditions, the predominant husbandry approach for Tibetan yaks involves dispersed family feeding, characterized by traditional grazing on grasses, straws, and lichens as their feed [20]. Effective grazing practices can avert supplemental feed consumption and contribute to the sustainability of the grazing system [21].

To survive the harsh conditions of extreme cold, low oxygen levels, and high altitudes, yaks need to consume more energy and develop more efficient metabolic mechanisms. Thus, both the host and gut microbes have developed unique physiological adaptations throughout evolution [22]. The gut microbiota not only aids the host in absorbing nutrients, metabolizing substances, and regulating the immune system but also facilitates the adaptation of the host to extreme environments. [23]. While yaks hold significant importance as domestic animals, the composition of their gut microbiota and their response to distinct plateau environments remain largely unexplored and undocumented. Additionally, few studies have examined whether alterations in the gut microbiota of yaks are linked to changes in altitudes and temperatures, especially captive yaks on the plateau [24]. Therefore, our study aimed to examine the bacterial and fungal compositions and diversities of gut microbiota in dry lot feeding Niangya yaks inhabiting various altitudes and temperatures employing high-throughput sequencing technology (HTST).

## 2. Materials and Methods

### 2.1. Sample Collection

About 100 fresh fecal samples (about 200 g each) from healthy dry lot feeding Niangya yaks were collected from three counties in Tibet Province, China: Damxung County (average annual temperature 1.3 °C, average altitude 4200 m), Linzhou County (average annual temperature 7.5 °C, average altitude 4200 m) and Nimu County (average annual temperature 6.7 °C, average altitude above 3800 m). To minimize potential contamination, fecal samples were collected twice from each yak. The collected fecal samples were promptly frozen using liquid nitrogen and then stored at −80 °C for subsequent analysis. In this study, we randomly selected six yaks each from Damxung County (DXF group), Linzhou County (LZF group), and Nimu County (NMF group), based on the county of sample collection. These samples were then sequenced and analyzed.

### 2.2. DNA Extraction and High-Throughput Sequencing

We extracted total microbial genomic DNA from 18 frozen fecal samples obtained from yaks in three distinct counties. Subsequently, we tested the purity and concentration of the DNA. Specific primers were employed for bacterial 16S rDNA (338F and 806R) and fungal ITS (ITS5F and ITS1R) genes along with barcode and high-fidelity DNA polymerase to amplify the V3/V4 hypervariable regions and ITS regions, respectively, based on the selected sequencing region [25]. Subsequently, we analyzed PCR products and excised and recovered the target fragments. After quantifying the recovered PCR amplification products based on the initial electrophoresis results, the proportions were adjusted to meet the sequencing specifications for each sample. After constructing the library, it underwent inspection to ensure quality before proceeding to the sequencing stage.

### 2.3. Bioinformatics and Functional Analysis

After trimming the raw data sequences, paired-end reads were filtered to remove low-quality sequences, followed by denoising, merging, detecting and removing chimera reads using DADA2 v1.20. Subsequently, the software outputted exemplar reads, ASV abundance tables, and a Venn map to determine the quantity of ASVs across the three groups. Alpha Diversity (α-diversity) analysis, which includes species accumulation box charts, species diversity curves, and various statistical analysis indexes, was utilized to evaluate the variances in microbial communities in terms of species richness and diversity in each sample, to judge the overall gut microbiome richness and diversity. Beta diversity (β-diversity) analysis, comprising Non-Metric Multi-Dimensional Scaling (NMDS), and Principal Coordinates Analysis (PCoA) was performed to compare microbial community compositions among different samples. LEfSe analysis was utilized to identify species characteristics that best elucidate differences between sample groups and quantify the inter-group influence of these characteristics. In the study, the prediction of bacterial and fungal functional abundances in yaks residing in diverse altitudes and temperatures was conducted using PICRUSt2. Additionally, KEGG function prediction analysis provided a valuable approach to investigating changes in metabolic functions within community samples in response to environmental fluctuations.

### 2.4. Statistical Analysis

Statistical analyses, including ANOVA, Chi-square tests, Kruskal–Wallis tests, and Dunn tests, were performed to assess differences in the data between the three groups using GraphPad Prism (v8.0) and SPSS (v26.0). Statistical significance was determined using a threshold of *p* < 0.05, indicating a level of significance within the analysis.

## 3. Results

### 3.1. Genomic Data Sequencing and Analysis

**Figure 1:**
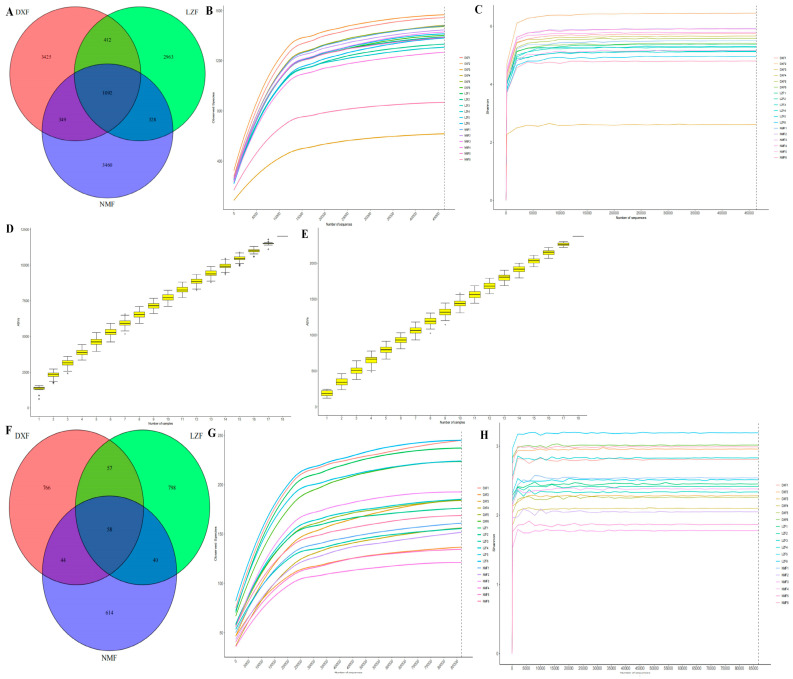
The distribution of ASVs and the quality of sequencing data. (**A**) Bacterial Venn map, (**B**) bacterial rarefaction curves, (**C**) bacterial Shannon curves, (**D**,**E**) bacterial and fungal species accumulation curves, (**F**) fungal Venn map, (**G**) fungal rarefaction curves, and (**H**) fungal Shannon curves.

Eighteen fecal samples were subjected to amplicon sequencing, with six samples collected from Damxung County, six from Nimu County, and six from Linzhou County. A total of 2,571,648 (DXF = 839,634, LZF = 859,286, NMF = 872,728) and 2,556,906 (DXF = 850,457, LZF = 845,708, NMF = 860,741) raw sequences were obtained from the V3/4 regions and ITS2 regions of targeted samples (Table 1). Following the optimization of raw data, we acquired a total of 2,453,629 and 2,384,997 filtered sequences of fungal and bacterial communities from three groups, respectively (Table 2). The Venn diagram revealed bacterial composition variations among the groups. Specifically, 5278, 4795, and 5229 ASVs were identified in the DXF, LZF, and NMF groups, respectively. Of these, 3425, 2963, and 3460 ASVs were unique to each group, while 1092 ASVs were shared across all three groups. Additionally, the DXF group and LZF group have 412 ASVs in common, the DXF group and NMF group have 349 ASVs in common, and the LZF group and NMF group have 328 ASVs in common (Figure 1A). The fungal composition among the groups was examined using a Venn diagram. It demonstrated that, in total, 925, 953, and 756 ASVs were identified in the DXF, LZF, and NMF groups, respectively. Among them, 766, 798, and 614 ASVs were unique to each group, while 58 ASVs were shared across all three groups (Figure 1F). The gradual flatting rarefaction curve indicated a reasonable amount of sequencing via randomly selecting only a certain sequencing amount of data from samples (Figure 1B,G). Shannon’s saturated tendency curves indicated that the sequencing depth and quantity adequately encompassed all species within the samples and indicated the necessity for additional investigation in this regard (Figure 1C,H). Meanwhile, the gradually smoothing species accumulation curves certified that the sample size is enough for data analysis (Figure 1D,E).

### 3.2. Gut Microbial Diversity Analysis

Alpha Diversity was used to estimate the microbiota variations within the community. Multiple α-diversity indexes describe the microbial diversity and richness, sequencing depth index, and phylogenetic diversity index. There is a positive correlation between α-diversity indexes and the diversity of samples. However, at the bacterial level, the variations of Chao1 (*p* = 0.23), observed_species (*p* = 0.18), Shannon (*p* = 0.14), ACE (*p* = 0.19), PD_whole_tree (*p* = 0.74) and goods_Coverage (*p* = 0.44) among yaks from different altitudes and temperatures were not statistically significant except Simpson (* *p* = 0.049) (Figure S1A). Meanwhile, variations of Chao1 (*p* = 0.071), observed_species (*p* = 0.089), Shannon (*p* = 0.331), Simpson (*p* = 0.423), ACE (*p* = 0.082), PD_whole_tree (*p* = 0.359), and goods_Coverage (*p* = 0.565) among three groups were all not statistically significant at the fungal level (Figure S1B).

Beta diversity analysis, such as principal coordinate analysis (PCoA) based on the weighted and unweighted_uniFrac of gut microbiota indicated that the DXF and NMF groups had distinct gut microbial community structures, while the LZF group exhibited a more similar microbial composition to the other two groups at the bacterial level (Figure S2A). Moreover, the distinct separation of dots representing the DXF group from the other two groups indicated that the fungal and gut bacterial structures of yaks were influenced by altitude and temperature to some extent (Figure S2C). In addition, non-metric multi-dimensional scaling (NMDS) could reflect the differences of samples via the distance between dots, and it could be seen that the stress of NMDS based on the unweighted and weighted_uniFrac is 0.1364, 0.0632, 0.1548 and 0.0705. At the bacterial and fungal level, respectively, these values were under 0.2 and the scatterplot revealed a notable separation between the data points corresponding to the DXF and NMF groups, which showed that the analysis results of NMDS were considered reliable and the environment would affect the gut bacterial community (Figure S2B,D).

### 3.3. Gut Bacterial Composition Analysis

**Figure 2:**
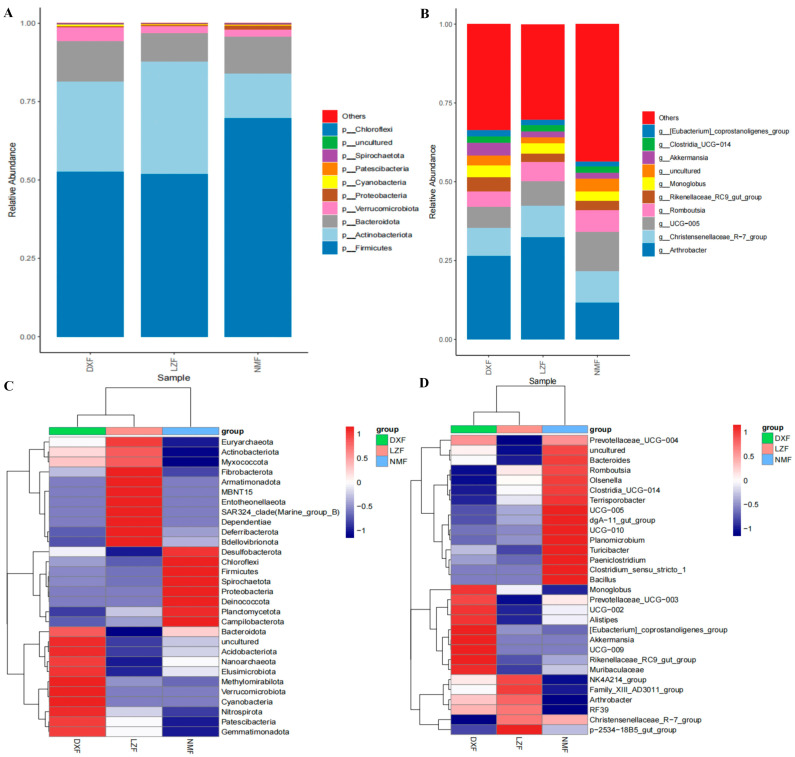
Variations in dominant gut bacterial taxa among yaks at different altitudes and temperatures. Gut bacterial composition on the phylum (**A**) and genus (**B**) levels. Clustered heatmap of yaks in different areas on the phylum (**C**) and genus (**D**) levels.

Marked variations in the taxonomic composition of dominant bacterial taxa were noted at both the phylum and genus levels among the three groups. We identified 36 phyla and 533 genera from the 18 fecal samples. Our investigation focused on the top 10 phyla and genera within the gut bacterial communities across the three groups (DXB, LZB, NMB groups) to find the differences in bacterial community. The phyla Firmicutes (DXF = 52.69%, LZF = 51.97%, NMF = 69.67%), Actinobacteriota (DXF = 28.66%, LZF = 35.77%, NMF = 14.23%) and Bacteroidota (DXF = 12.91%, LZF = 9.19%, NMF = 11.8%) were included. These three most abundant bacterial phyla accounted for 94.26%, 96.92%, and 95.74% of the total bacterial composition in the three groups, respectively. Other phyla such as Verrucomicrobiota (DXF = 4.42%, LZF = 2.15%, NMF = 2.09%), Proteobacteria (DXF = 0.25%, LZF = 0.26%, NMF = 0.14%), Cyanobacteria (DXF = 0.53%, LZF = 0.26%, NMF = 0.25%) and Patescibacteria (DXF = 0.23%, LZF = 0.16%, NMF = 0.1%) were comparatively less abundant (Figure 2A). Within the LZF group, the dominant genera were Arthrobacter (32.19%), Christensenellaceae_R-7_group (10.14%) and UCG-005 (7.77%). Notably, Arthrobacter constituted nearly one-third of the total bacterial composition at the genus level. However, the dominant genus in the NMF group was UCG-005 (12.39%), followed by Arthrobacter (11.58%) and Christensenellaceae_R-7_group (9.95%). The search results indicate that, in the DXF group, the dominant bacterial genera were Arthrobacter (26.38%), Christensenellaceae_R-7_group (8.94%), and UCG-005 (6.74%), constituting approximately 42.05% of all identified taxonomic groups in aggregate (Figure 2B). Additionally, the clustering heatmap analysis revealed variability in the gut bacterial community composition across the three groups, with differences observed in the relative abundances of dominant taxa at both the phylum (Figure 2C) and genus (Figure 2D) levels.

**Figure 3:**
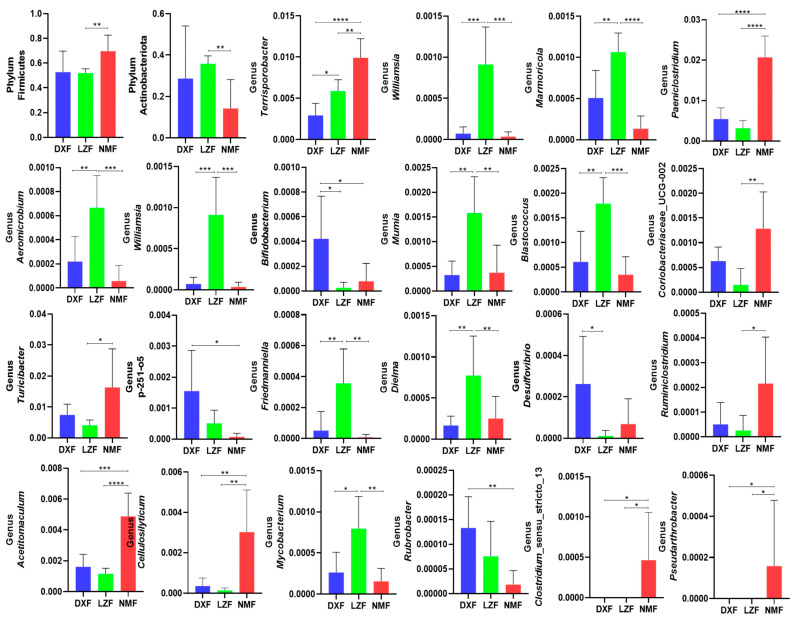
The gut bacterial comparisons among three groups. Metastatic analysis was employed to identify bacterial genera exhibiting significant differences in abundance. All data are presented as means ± standard deviation. * *p* < 0.05, ** *p* < 0.01, *** *p* < 0.001, **** *p* < 0.0001.

**Figure 4:**
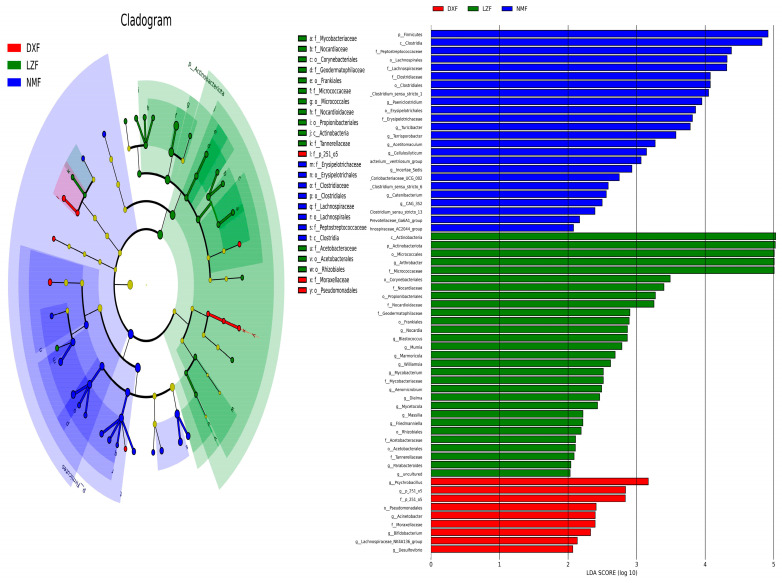
LEfSe analysis of gut bacteria. LDA scores > 2 was considered significantly different.

To analyze alterations in taxonomic composition among yaks inhabiting diverse altitudes and temperatures, metastatic analysis was conducted across various classification levels. At the phylum level, the dominance of Firmicutes was notably higher in the NMF group compared to both the DXF and LZF groups (*p* < 0.05 or *p* < 0.01) Besides, Actinobacteriota in the LZF group exhibited a dominant presence in contrast to the DXF and NMF groups. Additionally, significant differences in the dominant bacterial abundances among various groups were evident from comparisons at the genus level. Specifically, the prevalence of Bifidobacterium, Rubrobacter, and Desulfovibrio in the DXF group significantly exceeded that in the other two groups. Meanwhile, in the LZF group, Williamsia, Marmoricola, Aeromicrobium, Mumia, Blastococcus, Mycobacterium, and Dielma accounted for a larger proportion than others. Contrary to this, Terrisporobacter, Paeniclostridium, Acetitomaculum, Cellulosilyticum, Coriobacteriaceae_UCG-002, Turicibacter, and Ruminiclostridium played more important roles in NMF groups (Figure 3). Given the limitations of this discriminant analysis, it is possible that not all aspects of the taxonomic profile were detected. LEfSe analysis (LDA score > 2), known as biomarkers, was able to discern microorganisms, exhibiting higher relative abundances within each group than in the remaining groups. The results show that Pseudomonadales, p_251_o5, Desulfovibrio and nine biomarkers were detected in the DXF group, apart from the above-mentioned, which significantly differed, while Actinobacteriota, Micrococcales, Arthrobacter, and another 26 biomarkers were shown in the LZF group. In addition, the biomarkers in the NMF group were Peptostreptococcaceae, Clostridia, Clostridium_sensu_stricto_1, and so on (Figure 4).

### 3.4. Gut Fungal Composition Analysis

**Figure 5:**
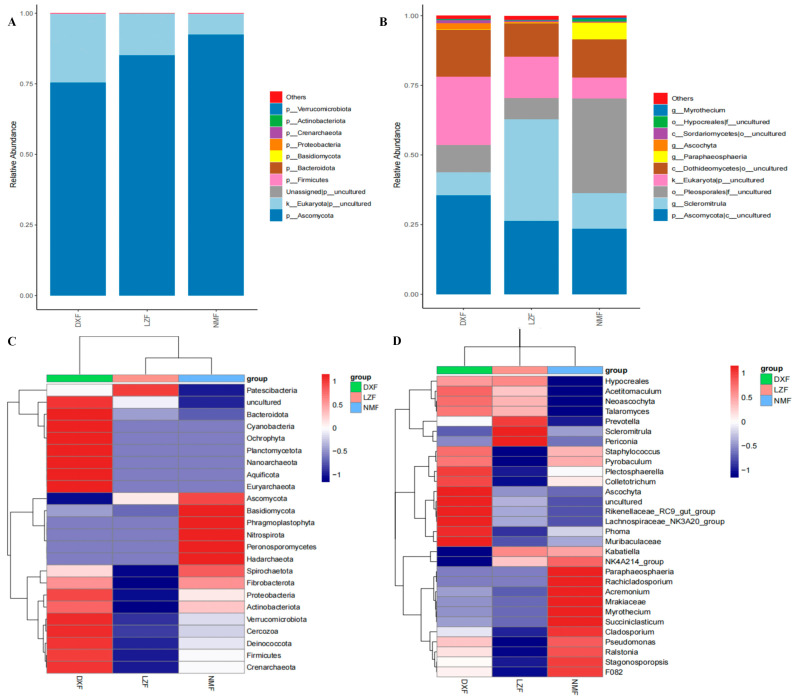
Variations in dominant gut fungal Taxas among Yaks at different altitudes and temperatures. Gut fungal composition on the phylum (**A**) and genus (**B**) levels. Phylum (**A**) and genus (**B**) levels. Clustered heatmap of yaks in different areas on the phylum (**C**) and genus (**D**) levels.

In this study, a total of 26 phyla and 153 genera were identified within the gut fungal community from 18 fecal samples. The phyla Ascomycota (DXF = 75.35%, LZF = 85.08%, and NMF = 92.42%) occupied the largest proportion; henceforward, the phyla uncultured (DXF = 24.52%, LZF = 14.82% and NMF = 7.42%) took second place. Other fungal phyla, including Firmicutes (DXF = 0.35%, LZF = 0.17%, and NMF = 0.26%), Bacteroidota (DXF = 0.03%, LZF = 0.02%, and NMF = 0.02%) and others in DXF, LZF, and NMF groups were identified in a low richness (Figure 5A). As illustrated in the clustered heatmap, the DXF group exhibited significantly higher abundances of the phyla Bacteroidota, Cyanobacteria, Ochrophyta, Planctomycetota, Nanoarchaeota, Aquificota, Euryarchaeota, Verrucomicrobiota, Cercozoa, Deinococcota, Firmicutes, and Crenarchaeota compared to the LZF and NMF groups. However, compared with DXF and NMF groups, the phyla Patescibacteria in the LZF group. Besides, the more fungal phyla in NMF group than other groups were Ascomycota, Basidiomycota, Phragmoplastophyta, Nitrospirota, Peronosporomycetes, Hadarchaeota (Figure 5C). Among the identified genera, Plectosphaerella, Colletotrichum, Ascochyta, Rikenellaceae_RC9_gut_group, Lachnospiraceae_NK3A20_group, Phoma, and Muribaculaceae in the DXF group accounted for more scale than other groups. The phyla Prevotella, Scleromitrula, and Periconia were significantly more in the LZF group; in addition, the richness of Paraphaeosphaeria, Rachicladosporium, Acremonium, Mrakiaceae, Myrothecium, and Succiniclasticum in the NMF group differed from DXF and NMF groups (Figure 5B,D).

**Figure 6:**
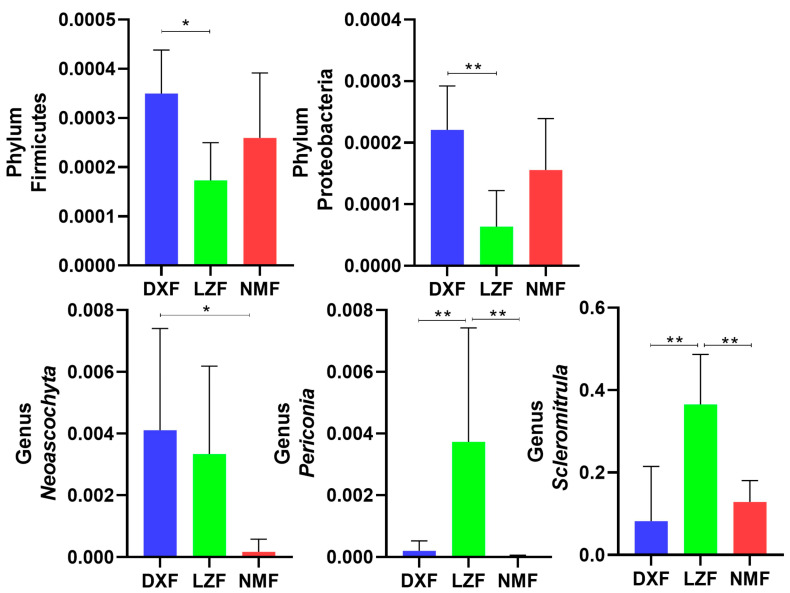
The gut fungal comparisons among three groups. Metastatic analysis was employed to identify bacterial genera exhibiting significant differences in abundance. All data are presented as means ±standard deviation. * *p* < 0.05, ** *p* < 0.01.

**Figure 7:**
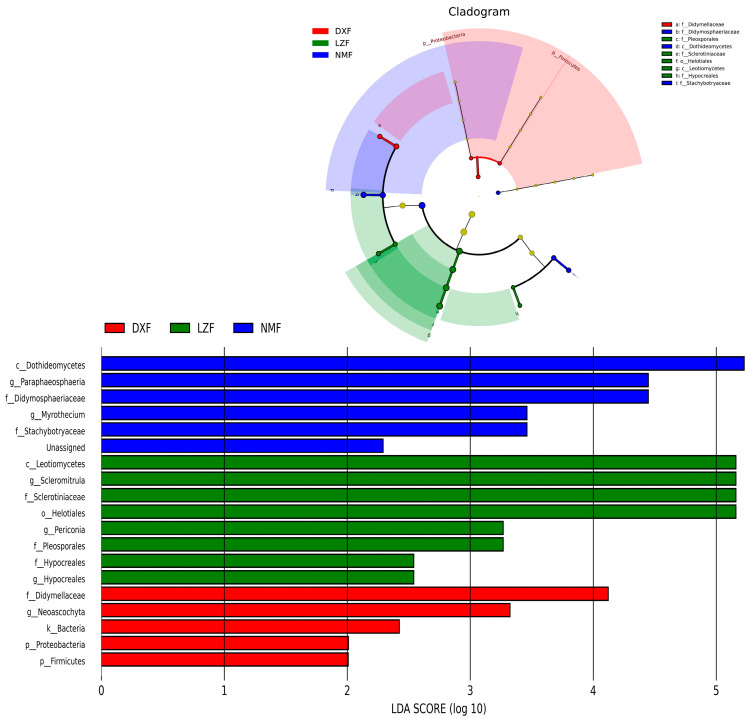
LEfSe analysis of gut fungi. LDA scores > 2 were considered significantly different.

Metagenomic analysis was employed to comparatively assess variations in the gut fungal community among the three groups. The DXF group exhibited a higher abundance of dominant phyla, particularly Firmicutes and Proteobacteria (* *p* < 0.05 or ** *p* < 0.01). At the genus level, the LZF group exhibited significantly higher richness of Scleromitrula and Periconia compared to the other two groups. However, Neoascochyta in the DXF group were more than others with significant differences (Figure 6). LEfSe analysis in conjunction with LDA scores was used to delve deeper into the alterations in gut fungal community composition (Figure 7).

### 3.5. Functional Predictive Analysis

**Figure 8:**
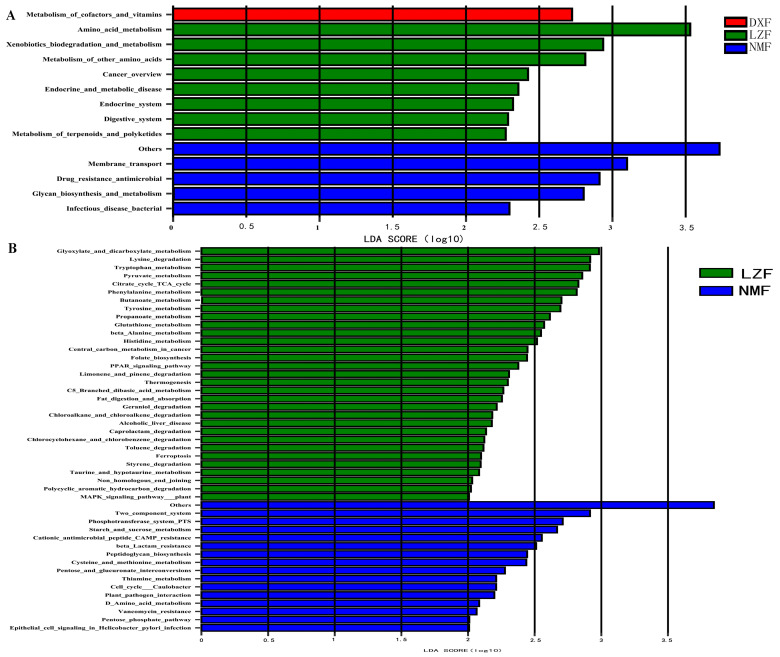
Functional biomarkers of gut bacteria in yaks. Functional pathways indicate variations in gut bacteria among yaks facing different temperatures and altitudes using the linear discriminant analysis effect size (LEfSe) plot. Functional pathways at KEGG level 2 (**A**) and KEGG level 3 (**B**) with a significant threshold were set at an LDA score of 2.0 (*p* < 0.05) among groups.

We utilized PICRUSt2 to analyze the functional pathways of the gut microbial community in the DXF, LZF, and NMF groups. Subsequently, the LEfSe method was employed to compare these pathways among the groups. While no significant differences were observed at KEGG level 1, notable disparities were detected at the deeper KEGG levels 2 and 3. In the prediction of bacterial function at KEGG level 2, there was a significant enrichment observed in the metabolism of cofactors and vitamins in the DXF group. In addition to this, eight functional pathways, including amino acid metabolism, xenobiotics biodegradation and metabolism, metabolism of other amino acids, cancer overview, endocrine and metabolic disease, endocrine system, digestive system, and metabolism of terpenoids and polyketides, observed notable enrichment in the LZF group. Moreover, there were five markedly rich functional pathways, including membrane transport, drug resistance antimicrobial, glycan biosynthesis and metabolism, infectious disease bacterial and others (Figure 8A). At KEGG level 3, the LZF group had 31 kinds of functional pathways with significantly increased differences like glyoxylate and dicarboxylate metabolism, lysine degradation, tryptophan metabolism, pyruvate metabolism, citrate cycle TCA cycle, folate biosynthesis, PPAR signaling pathway, thermogenesis and so on. Furthermore, there were 16 kinds of functional pathways (two-component system, the photransferase system PTS, starch and sucrose metabolism, cationic antimicrobial peptide CAMP resistance, beta Lactam resistance, peptidoglycan biosynthesis, cysteine and methionine metabolism, pentose and glucuronate interconversions, thiamine metabolism, cell cycle, Caulobacter, plant pathogen interaction, amino acid metabolism, vancomycin resistance, tipenove phospiate pathway, and epithelial cell signaling in Helicobacter pylori infection) in the NMF group; however, the DXF group had none (Figure 8B).

**Figure 9:**
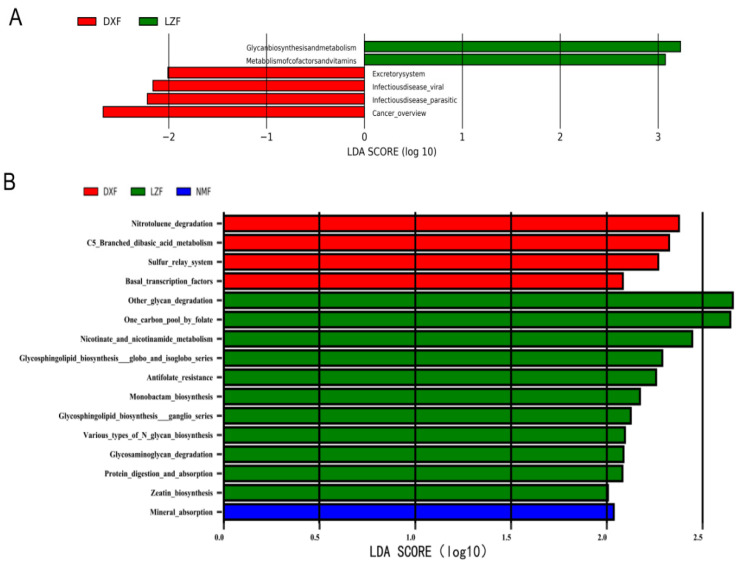
Functional biomarkers of gut fungi in yaks. Functional pathways indicate differences in gut fungi among yaks living in different temperatures and altitudes using the linear discriminant analysis effect size (LEfSe) plot. Functional pathways at KEGG level 2 (**A**) and KEGG level 3 (**B**) with a significant threshold were set at an LDA score of 2.0 (*p* < 0.05) among groups.

The functional pathways of fungi are equally important, the pathways in KEGG level 2, excretory system, infectious disease viral, infectious disease parasitic, and cancer overview were significantly enriched in the DXF group, and glycan bio synthesis and metabolism, metabolism of cofactors and vitamins were significantly upregulated in the LZF group, but the NMF group had nothing (Figure 9A). Functional pathways in KEGG level 3 showed that nitrotoluene degradation, C5 Branched dibasic acid metabolism, sulfur relay system, and basal transcription factors were significantly increased in the DXF group, while eleven functional pathways covering other glycan degradation, one carbon pool by folate, nicotinate and nicotinamide metabolism, glycosphingolipid biosynthesis globo and isoglobo series, antifolate resistance, monobactam biosynthesis, glycosphingolipid biosynthesis ganglion series, various types of N glycan biosynthesis, glycosaminoglycan degradation, protein digestion and absorption, and zeatin biosynthesis were enriched in the LZF group significantly; however, only one functional pathway was significantly increased, and it was mineral absorption (Figure 9B).

## 4. Discussion

Yaks residing at high altitudes are a vital source of livelihood and production, surviving in extreme environments characterized by low oxygen, low temperature, and intense ultraviolet radiation. Through long-term natural selection, yaks have developed adaptive characteristics in phenotype, physiology, and metabolism. The yak gut is a crucial organ for digestion, absorption, and immune functions. Gut microbes play a significant role not only in the digestion and absorption of feed, physiological processes, and disease development but also in the host’s adaptation to various geographical environments [26,27]. Environmental changes associated with different altitudes affect plant species and nutrient composition, thereby affecting the diversity, abundance composition, and function of gut microbes [22]. Moreover, ambient temperature can affect the adaptation of animals to the environment. The composition of intestinal core microflora and its metabolites exhibit significant variation under different temperatures. Extreme temperature mainly induces structural and functional differences in intestinal microflora, thus affecting the host phenotype [28].

Hence, it is crucial to investigate how altitude and temperature affect the composition and function of gut microbes in yaks. Fecal samples from three counties were analyzed for gut microbial adaptation to different altitudes and temperatures in Tibetan dry lot feeding yaks. The temperature in Damxung County (the average annual temperature is 1.3 °C) is significantly lower than in Linzhou County (the annual average temperature is 7.5 °C) and Nimu County (the average annual temperature is 6.7 °C); however, the altitude of Nimu County (the average altitude is above 3800 m) is lower than Damxung County (the average altitude is 4200 m) and Linzhou County (the average altitude is 4200 m).

This study utilized HTST to assess bacterial diversity among dry lot feeding yaks across varied environmental conditions. The findings indicated no significant difference in bacterial diversity between groups. This suggested that the diversity and richness of the bacterial community were similar across all groups, potentially attributed to minimal variations in altitude and temperature among the groups. However, PCoA and NMDS analyses of fungi could observe significant distances between the three groups, indicating differences in the distribution and structure of the intestinal fungi. Moreover, significant variations were observed in the microbiota structure across the samples. These findings suggest that alterations in environmental conditions significantly impact the abundance and structure of intestinal microbial composition in yaks.

Mammalian intestinal flora is a complex polymicrobial ecosystem, mainly composed of Firmicutes, Bacteroidetes, Actinobacteria, and Proteobacteria, providing host nutrition, promoting immunity, resisting pathogen colonization, and maintaining the stability of intestinal functions [29]. The dominant bacterial phyla were Firmicutes (DXF = 52.69%, LZF = 51.97%, NMF = 69.67%), Actinobacteriota (DXF = 28.66%, LZF = 35.77%, NMF = 14.23%) and Bacteroidota (DXF = 12.91%, LZF = 9.19%, NMF = 11.8%). The research results indicated that previous studies had found the relative abundance of Firmicutes to be higher than Bacteroidetes in the gut microbiota of grazing yaks [30]. This trend was also observed in the present study. Many researchers reported that Firmicutes and Bacteroidota were two primary bacterial phyla in the gut [31,32], but the second dominant bacterial phyla were Actinobacteriota instead of Bacteroidota, which did not match the present study. The phylum Firmicutes contains genes involved in energy metabolism and the breakdown of substances such as fiber and cellulose [33,34]. Actinobacteriota has been considered to be involved in the maintenance of gut barrier homeostasis, immune-modulation, and metabolism, and it serves as a potential probiotic that can resist a variety of drug-resistant pathogenic microorganisms [34,35]. Bacteroidota have the biological function of degrading proteins and carbohydrates [36]. In this study, the relative abundance of Actinobacteriota in LZF was significantly higher than in NMF, which indicated that the intestinal flora of yaks at high altitudes had significantly improved immunity against a variety of drug-resistant pathogenic microorganisms. Possibly, the abundance of Actinobacteria in the environment is relatively high [37,38], and the yaks may eat the soil when grazing grass, and the Actinobacteriota colonizes and grows in the gut of yaks. Additionally, the Firmicutes-to-Bacteroidota ratio is an important indicator to assess the effect of gut microbiota on host energy requirements. Considering the large temperature difference, the Firmicutes-to- Bacteroidota ratio of NMF (mean temperature is −40 °C in January and 15 °C in July) was distinctly higher than LZF (mean temperature is −5.4 °C in January and 14 °C in July), which suggested that the yak gut bacteria have an enhanced energy metabolism and a catabolism-related capacity to counteract the large temperature difference. In addition, Proteobacteria is a kind of common gut bacteria with strong adaptability and potential pathogenicity, and the increased abundance of Proteobacteria serves as an important feature of intestinal dysbiosis [39,40]. In this study, the relative abundance of Proteobacteria (the content is very low as a percentage of total microbiota) in DXF was significantly higher than LZF, which indicated that low temperature may lead to the increased abundance of Proteobacteria, thus affecting host health negatively. Gut fungi play an important role in maintaining intestinal homeostasis, preventing infection, and serving as reservoirs for opportunistic microorganisms. The abundances of the dominant fungal phyla were Ascomycota (DXF = 75.35%, LZF = 85.08%, and NMF = 92.42%), but there were no significant differences between groups.

We used metastatic analysis to check the variations in the gut microbiota (bacteria and fungi) among three groups and found significant variations in some bacterial and fungal taxa of yaks in different altitudes and temperatures. Among some bacterial and fungal taxa in different groups, these taxa are likely to play an important role in gut microbial homeostasis and intestinal function. The growth of bacteria with significant differences is not only beneficial bacteria (Bifidobacteria) but also pathogenic bacteria (Desulfovibrio). Bifidobacteria possess the ability to utilize a wide range of dietary carbohydrates that are not fully degraded in the upper parts of the intestine, and exopolysaccharides (EPSs) isolated from Bifidobacteria possess probiotic function, antioxidant, anticancer, and immunomodulatory [41,42,43]. Desulfovibrio is a common symbiotic bacterium in the gastrointestinal tract. Although its abundance is relatively low in a healthy gut, it is an opportunistic pathogen that can proliferate excessively in the presence of disease [44,45]. Rubrobacter can also be detected clearly in a very low proportion in the DXF group, but Rubrobacter exists in soil commonly, which possesses key chlorophyll-biosynthesis-related proteins to have photosynthesis [46]. Amazingly, we detected a variety of genera of the phylum Actinobacteriota, including Williamsia, Marmoricola, Aeromicrobium, Mycobacterium, Mumia, and Blastococcus, which were significantly enriched in the LZF group. Many researchers reported that Williamsia leads to a decrease in immunity, which can trigger infections [47,48]. Marmoricola often isolated from soil can be considered as a biological indicator of soil pollution [49,50]. Aeromicrobium exhibits the potential to combat plant diseases through the production of antimicrobial molecules. [51]. Mycobacterium is a facultative intracellular bacterium that induces inflammation and cell necrosis [52]. Kumar et al. found dielma in the infected group with higher LDA scores in the gut microbiome of Labeo rohita, and Ankjaer et al. reported that dielma isolated from human fecal samples will increase the likelihood of bacteremia [53,54]. As one of the four main categories of intestinal flora, the maintenance of intestinal equilibrium relies significantly on the contribution of Actinobacteriota, although their proportion is not large. In addition, Actinobacteriota also participates in the decomposition of plant-derived carbohydrates and the body’s immune inflammation and autoimmune reactions [55]. YuJia et al. found that the high level of actinomycetes in the tadpole intestinal tract is conducive to the maintenance of intestinal homeostasis and the absorption of nutrients from plants [56]. Ruminant symbiotic microorganisms have rich species diversity and can co-evolve with bacteria, actinomycetes, and fungi for a long time to establish a variety of symbiotic relationships, thus affecting the nutrition, metabolism, growth, development, lifespan, and evolution of the host [57]. Given that there are few studies on Actinobacteriota in the intestinal tract of ruminants, and the mechanism of action on the intestinal tract needs further study, we speculated that yak grazing will increase the probability of yaks eating soil enriched in Actinobacteriota, resulting in the colonization of Actinobacteriota in the intestinal tract of yaks. To adapt to the grazing environment at high altitudes, Actinobacteriota, and other bacteria gradually build a balance and jointly promote intestinal homeostasis and the development of yaks.

Notably, several bacterial genera regarded as beneficial to intestinal health, including Terrisporobacter, Acetitomaculum, Coriobacteriaceae_UCG-002, Turicibacter, and Ruminiclostridium, were significantly elevated in the NMF group. These bacteria play crucial roles in digestion and absorption, substance metabolism, and immune enhancement. Terrisporobacter can be used to produce lactic acid and acetic acid, some micro-organisms in the intestine can be used to synthesize butyric acid from acetic acid and lactic acid, and Acetitomaculum produces butyric acid as well [58], the production of which increases the energy supply [59,60]. Cellulosilyticum has previously been shown to be a cellulose-decomposing bacterium that can use a variety of natural cellulose as the sole carbon source, playing a key role in feed digestion [61]. Coriobacteriaceae_UCG-002 is positively correlated with Glycerophosphorylcholine, which has certain functions to promote lipid metabolism, reduce the local accumulation of lipids and promote glucose homeostasis, as well as bile acids [62]. Turicibacter serves as gut probiotics, which has been demonstrated to be linked with immune-enhancing effects, and modifies host bile acids and lipid metabolism [63,64]. Ruminiclostridium uses ABC transporters regulated by the two-component system (TCS) to absorb extracellular sugars to efficiently degrade cellulose [65]. However, Paeniclostridium is involved in enteric and histotoxic infections in a variety of animals [66]. Yaks, as strictly herbivorous ruminants, need to consume a large amount of forage to maintain their energy consumption and growth under the harsh, high-altitude environment of the Qinghai–Tibetan Plateau. Based on the results, it is hypothesized that the average altitude of the Qinghai–Tibet Plateau, exceeding 3000 m, is not positively correlated with the abundance of beneficial bacteria. This may be attributed to the relatively high crude protein and crude fat content of pasture grasses at lower elevations. The high altitude (over 4200 m) is not conducive to the growth of beneficial bacteria, but the medium altitude is not only conducive to the growth of beneficial bacteria, but also increases the F/B ratio, promoting digestion and absorption and ensuring the energy supply of yaks.

Fungi are ubiquitous organisms with a wide distribution in almost all ecosystems [67]. Intestinal fungi are an integral part of the gut microbiota. Scleromitrula and Neoascochyta are necrotic fungi with a narrow host range and have negative effects on plants [68,69]. Members of the genus Periconia are commonly found in plants or soil with notable antioxidant and antibacterial activities [70]. Only the abundance of the above individual fungi varied significantly between groups, so we thought altitude and temperature had no significant effect on fungi. However, there were few reports on their occurrence in the gut, indicating that the composition and abundance of yak fungi still need further study.

Studies investigated the functional pathways of the intestinal microbial communities in yaks living in Damxung County, where the altitude is high and the temperature is low. The analysis revealed significant enrichment of certain functional pathways at the KEGG level 2, including the metabolism of cofactors and vitamins, excretory system, and infectious disease (viral and parasitic). Additionally, amino acid metabolism, xenobiotics biodegradation, the metabolism of other amino acids, endocrine and metabolic disease, the endocrine system, the digestive system, the metabolism of terpenoids and polyketides, glycan biosynthesis and metabolism, the metabolism of cofactors and vitamins were upregulated in yaks living at high altitude and in the higher temperatures of Linzhou County. Most of the functions are related to metabolism, combined with the high abundance of Actinobacteriota. A previous study showed that the intestinal Actinobacteriota of giant panda had the potential to degrade cellulose and play a role in carbohydrate and amino acid metabolism [71], so we speculated that Actinobacteriota may be related to body metabolism. Furthermore, membrane transport, drug resistance antimicrobial, glycan biosynthesis and metabolism, infectious disease bacterial, and others were significantly enhanced in the intestinal microbiota of yaks living in Nimu County with lower altitudes and high temperatures. Functional biomarkers correlated with microbial biomarkers by correlation comparison, in general, promoting cellulose degradation and material metabolism and increasing energy supply for yaks living in Nimu County. At KEGG level 3, the DXF group exhibited only a few important functional biomarkers within the intestinal microbiota, whereas the LZF group demonstrated up to 40 distinct biomarkers. Most of these biomarkers were related to metabolism, biosynthesis, and substance degradation, which positively correlated with the findings at KEGG level 2. This indicates that the intestinal microbiota of yaks in Linzhou County is advantageous for nutrient absorption and substance metabolism. Moreover, in the NMF group, in addition to metabolic and biosynthetic functions, the two-component system and drug resistance were found to be obvious. The two-component system (TCS) plays a key role in the survival of a variety of bacteria, it plays an important role in bacterial homeostasis, and it can also activate them to produce multiple-antibiotic resistance [72]; however, determining whether altitude and temperature affect the resistance of yaks still needs to be combined with the actual feeding plan.

## 5. Conclusions

To sum up, previous studies reported the microbial composition of free-ranged plateau animals [73,74,75], while this study analyzed changes in intestinal bacterial and fungal communities in the dry lot feeding yaks living at different altitudes and temperatures. The results reveal subtle differences in the diversity of intestinal bacteria and fungi, but such differences are accompanied by significant changes in taxonomic composition. In particular, different from previous studies, the abundance of Actinobacteriota ranked second in this study; amino acid metabolism and biosynthesis were the dominant functional biomarkers through functional pathway prediction analysis, indicating that Actinobacteriota had potential probiotic effects on yaks. This study advanced the understanding of yak gut microbiota across diverse habitats, highlighting bacterial and fungal composition as key factors influencing yak adaptation to varying altitudes and temperatures. Moreover, the findings may contribute to establishing a theoretical framework for assessing yak environmental adaptability through the gut microbe’s perspectives.

## Figures and Tables

**Table 1:** The bacterial sequence information of yaks in group DXF, LZF, and NMF.

Sample	Input	Filtered	Percentage of Input Passed Filter	Denoised	Merged	Percentage of Input Merged	Non-Chimeric	Percentage of Input Non-Chimeric
DXF1	136082	130346	95.78	120718	80331	59.03	53182	39.08
DXF2	141104	134633	95.41	124845	75867	53.77	46342	32.84
DXF3	136664	129770	94.96	125961	110875	81.13	76494	55.97
DXF4	146079	139693	95.63	130785	91788	62.83	65521	44.85
DXF5	136149	130234	95.66	121562	84270	61.9	60881	44.72
DXF6	143556	137327	95.66	129426	93680	65.26	64440	44.89
LZF1	136495	130353	95.5	122882	88264	64.66	61717	45.22
LZF2	148617	141799	95.41	134871	100535	67.65	69192	46.56
LZF3	144930	138497	95.56	129931	92348	63.72	62404	43.06
LZF4	142995	136314	95.33	128517	92418	64.63	65027	45.48
LZF5	137609	131661	95.68	123131	87433	63.54	56443	41.02
LZF6	148640	141526	95.21	133762	100860	67.86	69871	47.01
NMF1	142836	135583	94.92	126127	90361	63.26	64030	44.83
NMF2	149456	142494	95.34	133232	92148	61.66	60494	40.48
NMF3	149132	142325	95.44	133581	99781	66.91	71098	47.67
NMF4	144597	137914	95.38	129975	93392	64.59	65198	45.09
NMF5	144850	137844	95.16	128590	90033	62.16	63314	43.71
NMF6	141857	135316	95.39	128940	95302	67.18	60338	42.53

**Table 2:** The fungal sequence information of yaks in group DXF, LZF, and NMF.

Sample	Input	Filtered	Percentage of Input Passed Filter	Denoised	Merged	Percentage of Input Merged	Non-Chimeric	Percentage of Input Non-Chimeric
DXF1	144836	134999	93.21	132574	123581	85.32	116040	80.12
DXF2	147173	137090	93.15	134980	123723	84.07	122130	82.98
DXF3	143732	137507	95.67	135999	131144	91.24	102926	71.61
DXF4	137044	130446	95.19	129048	121427	88.6	105729	77.15
DXF5	139231	131506	94.45	129800	120644	86.65	113252	81.34
DXF6	138441	133192	96.21	131755	126352	91.27	106313	76.79
LZF1	137122	127128	92.71	124315	106359	77.57	98881	72.11
LZF2	135112	126010	93.26	123606	111180	82.29	102463	75.84
LZF3	143958	134029	93.1	131588	114105	79.26	107885	74.94
LZF4	140049	128155	91.51	125217	106839	76.29	98141	70.08
LZF5	141776	130590	92.11	126389	103262	72.83	95692	67.5
LZF6	147691	134614	91.15	131005	111582	75.55	97802	66.22
NMF1	141888	125924	88.75	122186	95760	67.49	89502	63.08
NMF2	147307	140830	95.6	139462	129825	88.13	117810	79.98
NMF3	148348	135519	91.35	132111	113317	76.39	104332	70.33
NMF4	144240	134496	93.24	132665	126219	87.51	104606	72.52
NMF5	138385	130511	94.31	129528	125005	90.33	120755	87.26
NMF6	140573	132451	94.22	130433	121311	86.3	86740	61.7

## Data Availability

The raw data in this study were deposited in the NCBI database under accession number: PRJNA1097828.

## Supplementary Materials

The following supporting information can be downloaded at: https://www.mdpi.com/article/10.3390/microorganisms12071350/s1, Figure S1: Alpha diversity index analysis of yaks living in different altitudes and temperatures. (A) Bacterial, (B) Fungal. Data were represented as means ± SD (n = 6). * *p* < 0.05; Figure S2: Different altitudes and temperatures changed intestine bacterial and fungal beta diversity in yaks. (A) PCoA plots of bacteria based on the unweighted and weighted_UniFrac distance, (B) NMDS plots of bacteria based on the weighted and unweighted UniFrac distance. (C) PCoA plots of fungi based on the unweighted and weighted_UniFrac distance, (D) NMDS plots of fungi based on the unweighted and weighted UniFrac distance.
