# Supplementary material for: Gut Microbial Adaptation to Varied Altitudes and Temperatures in Tibetan Plateau Yaks
Source: Microorganisms. 2024 Jul 1;12(7):1350. doi: 10.3390/microorganisms12071350 (PMC11278572; doi:10.3390/microorganisms12071350)
Supplement: Supplementary file 1 [file microorganisms-12-01350-s001.zip › microorganisms-3030057-supplementary.pdf]

Supplementary Materials:

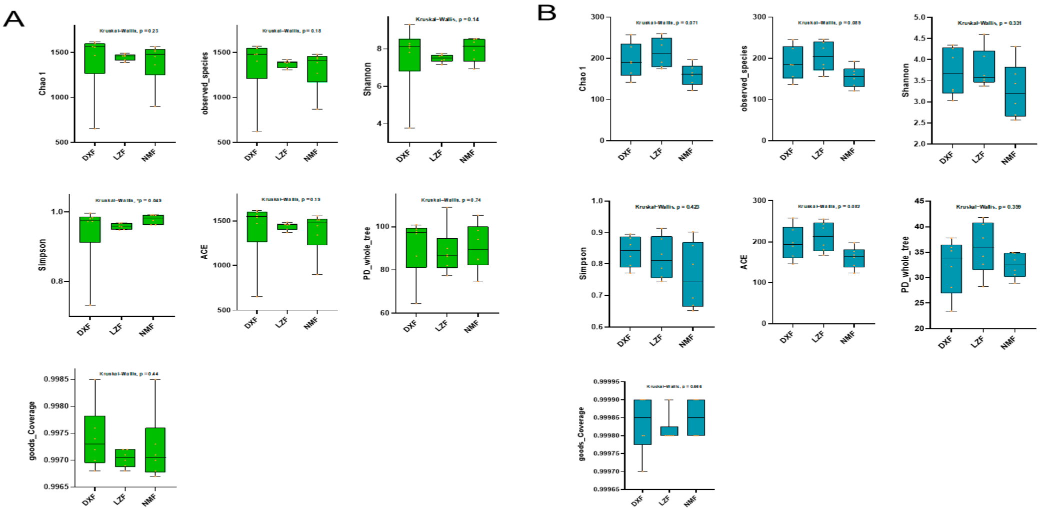

Figure S1. Alpha diversity index analysis of yaks living in different altitudes and temperatures. (A) Bacterial, (B) Fungal. Data were represented as means ± SD (n=6). *P＜0.05.

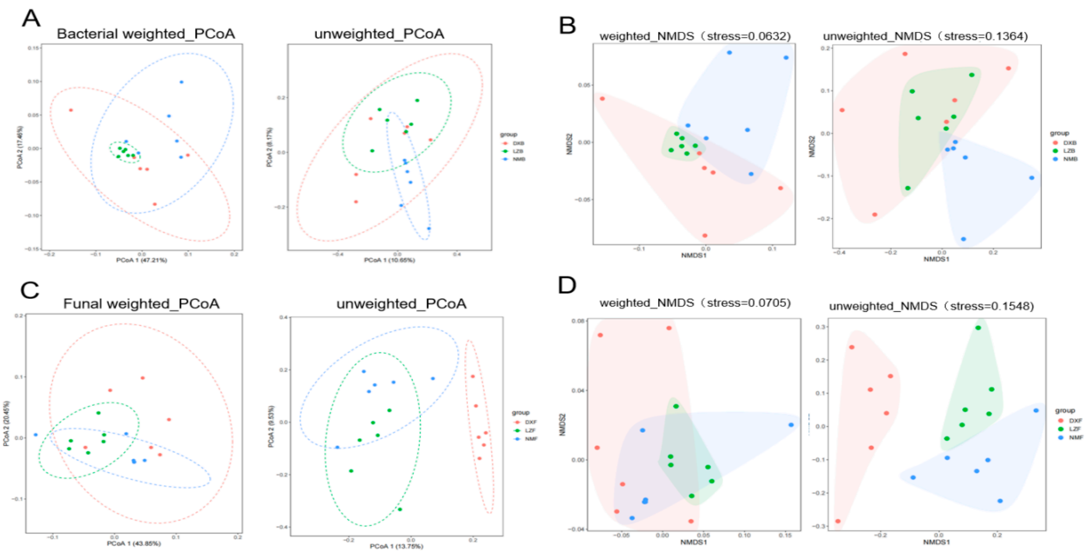

Figure S2. Different altitudes and temperatures changed intestine bacterial and fungal beta diversity in yaks. (A) PCoA plots of bacteria based on the unweighted and weighted_UniFrac distance, (B) NMDS plots of bacteria based on the weighted and unweighted UniFrac distance. (C) PCoA plots of fungi based on the unweighted and weighted_UniFrac distance, (D) NMDS plots of fungi based on the unweighted and weighted UniFrac distance.
